# 

**Published:** 1883-08

**Authors:** 


					﻿VOL.JTI.
AUGUST, 1883.
NO. 8.
THE
HOMEOPATHIC pHY^lClAK
A MONTHLY JOURNAL OF MEDICAL SCIENCE.
“ Magna est verilas, et prcevalebit.”
ZHL CT. LJEHL IZE. 3D.,
EDITOR.
CONTENTS:
(See inside of Cover.}
PHILADELPHIA:
No. 3109 CHESTNUT STREET.
2ST 33 'W YORK?
A. L. CHATTERTON, PUBLISHING COMPANY, Publisher’s^Agents.
LONDON:'
ALFRED HEATH & CO., Sole Agents FOR Great Britain,
114 Ebury Street,' Eaton Square.
Yearly Subscription, $2.50, invariably in advance.
Entered at the Philadelphia Post-Office as Second-Class Mail Matter.
				

